# Supplementary material for: Proximity labeling of DAF-16 FOXO highlights aging regulatory proteins
Source: Nat Commun. 2025 Dec 11;16:11355. doi: 10.1038/s41467-025-66409-0 (PMC12727705; doi:10.1038/s41467-025-66409-0)
Supplement: Supplementary file 1 — Supplementary Information [file 41467_2025_66409_MOESM1_ESM.pdf]

Supplementary Information

Proximity labeling of DAF-16 FOXO highlights aging regulatory proteins

Murat Artan, Hanna Schoen, Mario de Bono

Supplementary Figures 1 to 6

Supplementary Tables 1 to 7

Supplementary Fig. 1

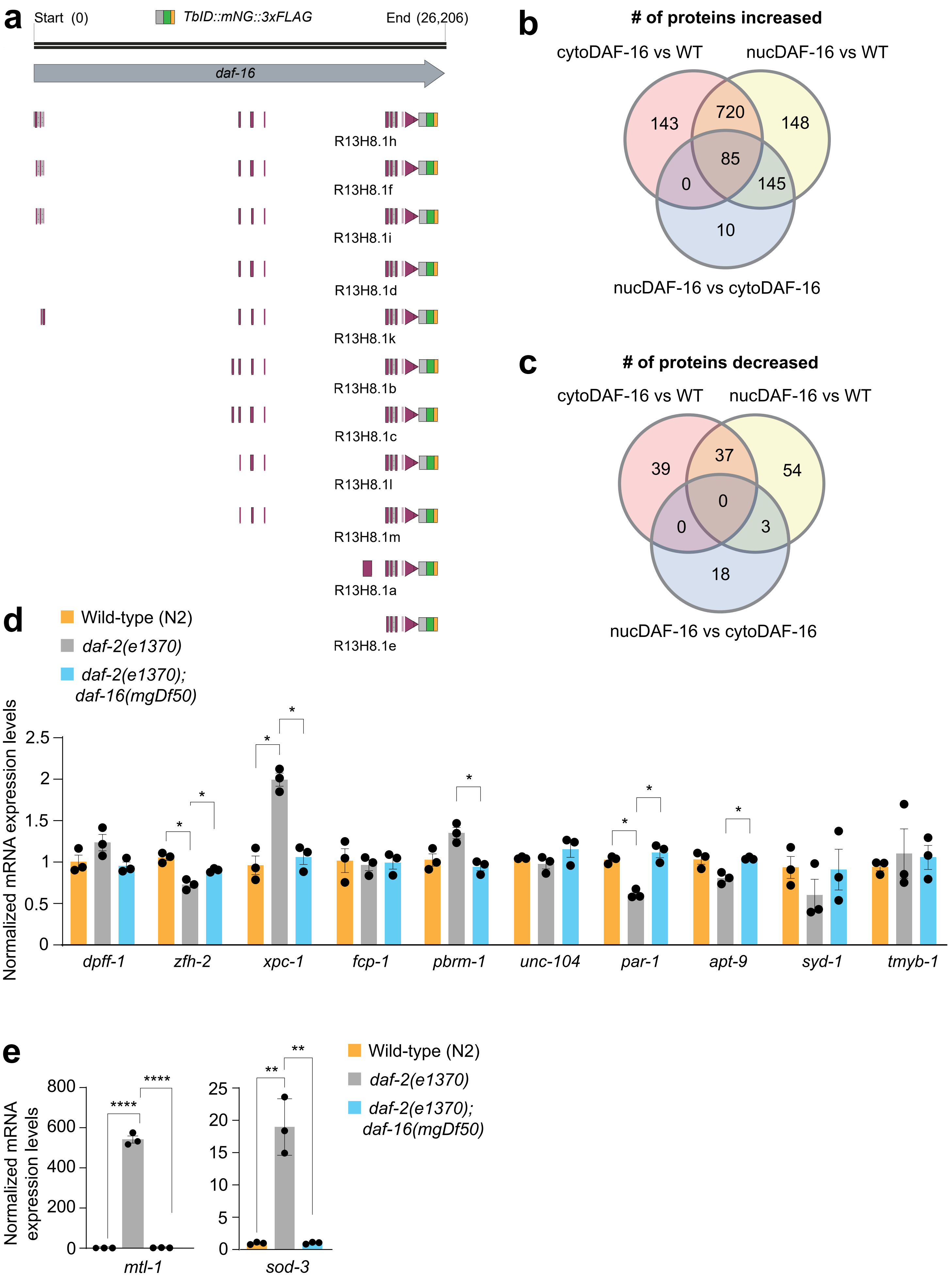

Supplementary Fig. 1

a, Schematic showing all known or predicted *daf-16* isoforms (labeled R13H8.1a-m) and highlighting the knock-in site of the TurboID::mNG::3xFLAG cassette just upstream of the *daf-16* stop codon.

b, c, Venn diagram showing proteins whose levels increase (**b**) or decrease (**c**) with a $\log_2 \geq 2$ for the following comparisons: cytoDAF-16 vs WT, nucDAF-16 vs WT and nucDAF-16 vs cytoDAF-16.

d, qRT-PCR analysis of selected potential DAF-16 cytosolic or nuclear interactors. Experiments were performed on total RNA isolated from wild-type (N2), *daf-2(e1370)* or *daf-2(e1370); daf-16(mgDf50)* animals. n=3 independent biological repeats. Error bars represent s.e.m; $^{*}p < 0.05$, two-tailed unpaired *t*-test with Benjamini-Hochberg correction.

e, qRT-PCR analysis of the DAF-16 transcriptional target genes *sod-3* and *mtl-1*. Experiments were performed on total RNA isolated from wild-type (N2), *daf-2(e1370)* or *daf-2(e1370); daf-16(mgDf50)* animals. This panel is a positive control for the experiments performed in panel **d**. n=3 independent biological repeats. Error bars represent s.e.m; $^{**}p < 0.01$, $^{****}p < 0.0001$, two-tailed unpaired *t*-test with Benjamini-Hochberg correction.

Supplementary Fig. 2

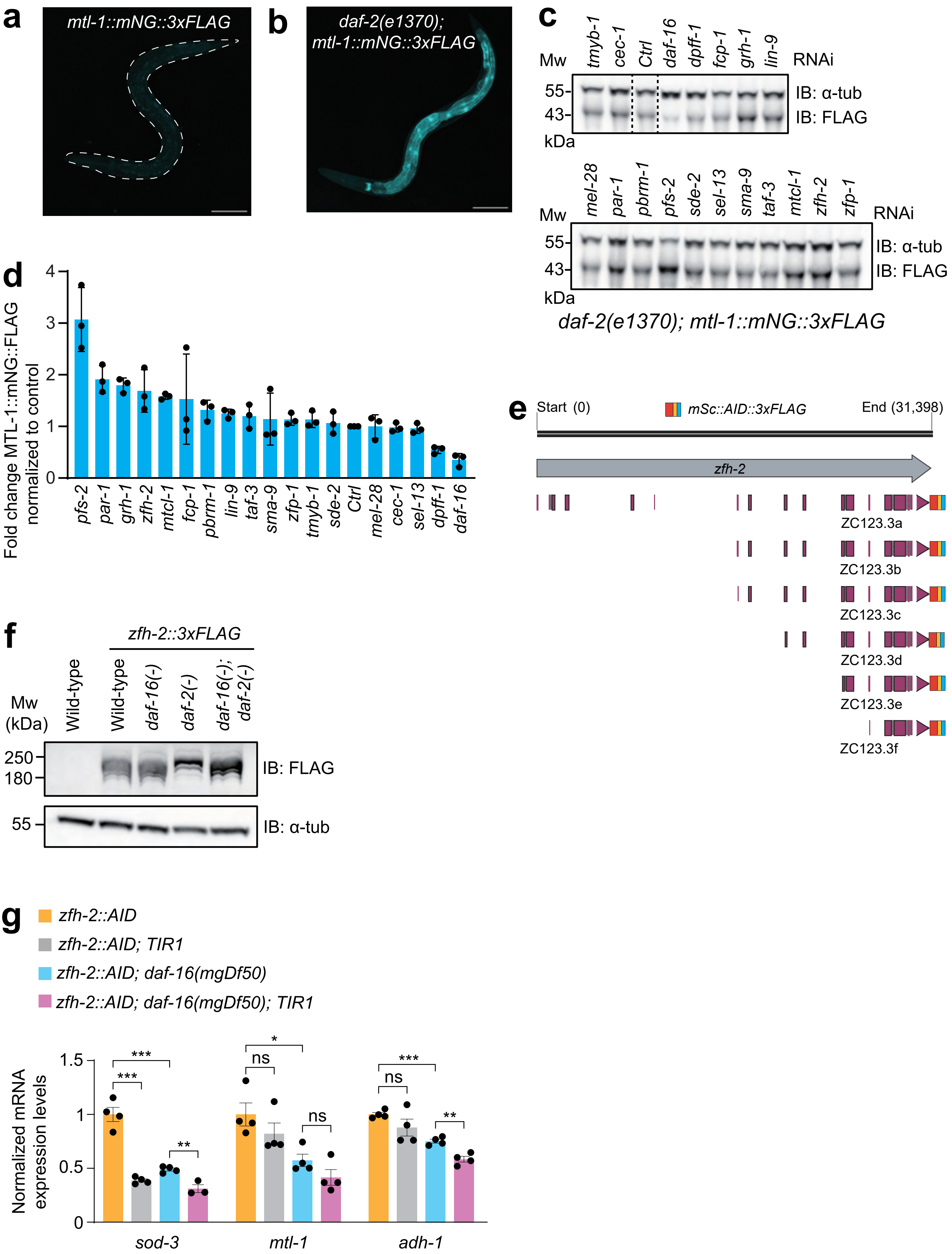

Supplementary Fig. 2. Secondary RNAi screen of putative DAF-16 interactors in a *daf-2* background.

a, b, Confocal images of gene-edited animals expressing MTL-1:: mNG::3xFLAG in a wild-type (**a**) and *daf-2(e1370)* (**b**) mutant background. Scale bar: 100 µm.

c, Western blots showing levels of FLAG-tagged MTL-1 following RNAi knockdown of putative DAF-16 interactors in a *daf-2* mutant background. *daf-16* RNAi and empty vector (Ctrl) provide controls. α-tubulin provides a loading control.

d, Quantification of MTL-1-FLAG band intensity as in panel **c** normalized first to the α-tubulin loading control and then to the empty vector RNAi control (n=3 independent biological repeats).

e, Schematic showing all known or predicted *zfh-2* isoforms (labelled ZC123.3a-f) and the knock-in site of an mSc::AID::3xFLAG cassette, just upstream of the *zfh-2* stop codon.

f, Western blot showing various isoforms of endogenously tagged ZFH-2::mSc::AID::3xFLAG in wild-type, *daf-16(mgDf50)*, *daf-2(e1370)* and *daf-16(mgDf50); daf-2(e1370)* backgrounds. n=3 independent biological repeats with similar results.

g, RT-qPCR analysis of the DAF-16 transcriptional target genes *sod-3*, *mtl-1* and *adh-1*. Experiments were performed on total RNA isolated from control or ZFH-2-depleted animals in a wild-type or *daf-16(mgDf50)* mutant background. Animals were grown on NGM plates until the late L3 stage and then transferred to auxin-containing NGM plates for 12 hours prior to harvesting RNA (n=4 independent biological repeats). Error bars represent s.e.m; ns, not significant, $*p < 0.05$, $**p < 0.01$, $***p <$

0.001, two-tailed unpaired *t*-test with Benjamini-Hochberg correction

Source data are provided as a Source Data file.

Supplementary Fig. 3

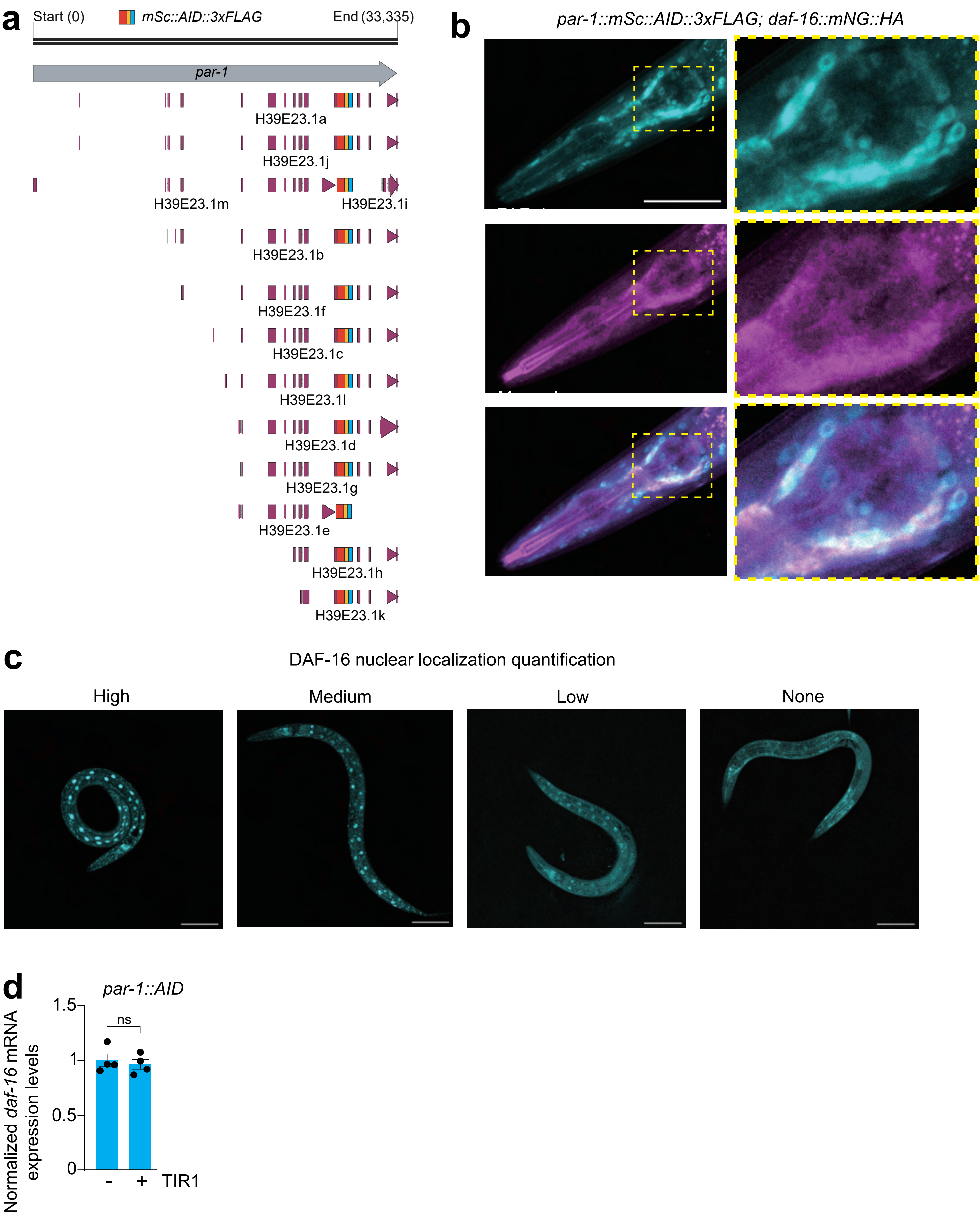

Supplementary Fig. 3. Tagging PAR-1 by CRISPR gene editing

a, Schematic showing all known or predicted *par-1* isoforms (labeled as H39E23.1a-m) and highlighting the knock-in site used to insert an mSc::AID::3xFLAG cassette to knock down all 13 PAR-1 isoforms. The cassette was inserted using gene-editing into the 11th exon (between A895 and A896 in isoform a).

b, Confocal images of animals expressing gene-edited PAR-1::mSc::AID::3xFLAG and DAF-16::mNG::HA. There is overlapping expression in hypodermal and neuronal tissues. Zoomed area shows the nerve ring, a region of dense neuropil rich in synapses. Scale bar: 50 μm.

c, Images highlighting semi-quantitative scoring of DAF-16::mNG nuclear localization. Scale bars: 100 μm.

d, RT-qPCR analysis of *daf-16* mRNA levels. Experiments were performed in control or PAR-1-depleted animals. Animals were grown on NGM plates until the late L3 stage and then transferred to auxin-containing NGM plates for 12 hours prior to harvesting for RNA extraction (n=4 independent biological repeats). Error bars represent s.e.m; ns: not significant, two-tailed unpaired *t*-test with Benjamini-Hochberg correction.

Supplementary Fig. 4

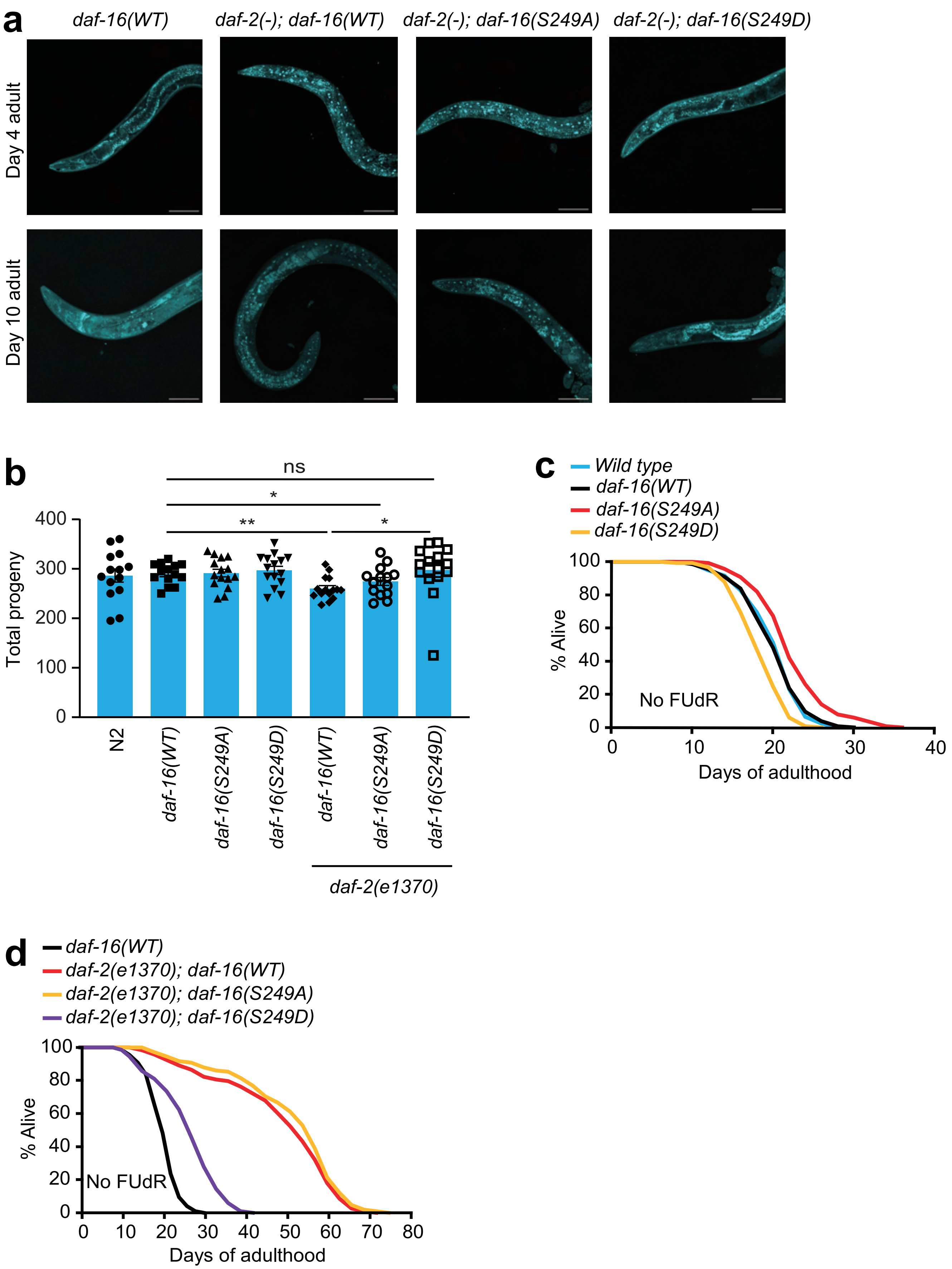

Supplementary Fig. 4. S249D or S249A substitutions alter DAF-16 subcellular localization and lifespan

a, Confocal microscopy images of DAF-16 nuclear localization in *daf-16(WT)::mNG*, *daf-2(e1370); daf-16(WT)::mNG, daf-2(e1370); daf-16(S249A)::mNG* or *daf-2(e1370); daf-16(S249D)::mNG* transgenic animals at the fourth or tenth day of adulthood.

b, Brood size of *daf-16(WT)::mNG*, *daf-16(S249A)::mNG* and *daf-16(S249D)::mNG* gene-edited animals in wild-type or *daf-2(e1370)* backgrounds. Error bars represent s.e.m; ns, not significant, *$p < 0.05$, **$p < 0.01$, Welch's unequal variances *t*-test.

c, Lifespan analysis of wild-type (N2), *daf-16(WT)::mNG*, *daf-16(S249A)::mNG* or *daf-16(S249D)::mNG* transgenic animals without FUdR supplementation.

d, Lifespan analysis of *daf-16(WT)::mNG*, *daf-2(e1370); daf-16(WT)::mNG*, *daf-2(e1370); daf-16(S249A)::mNG* or *daf-2(e1370); daf-16(S249D)::mNG* transgenic animals without FUdR supplementation. The *daf-16(WT)::mNG* data are also used in panel **c**.

See Supplementary Table 4 for values and statistical analyses for lifespan assays.

Supplementary Fig. 5

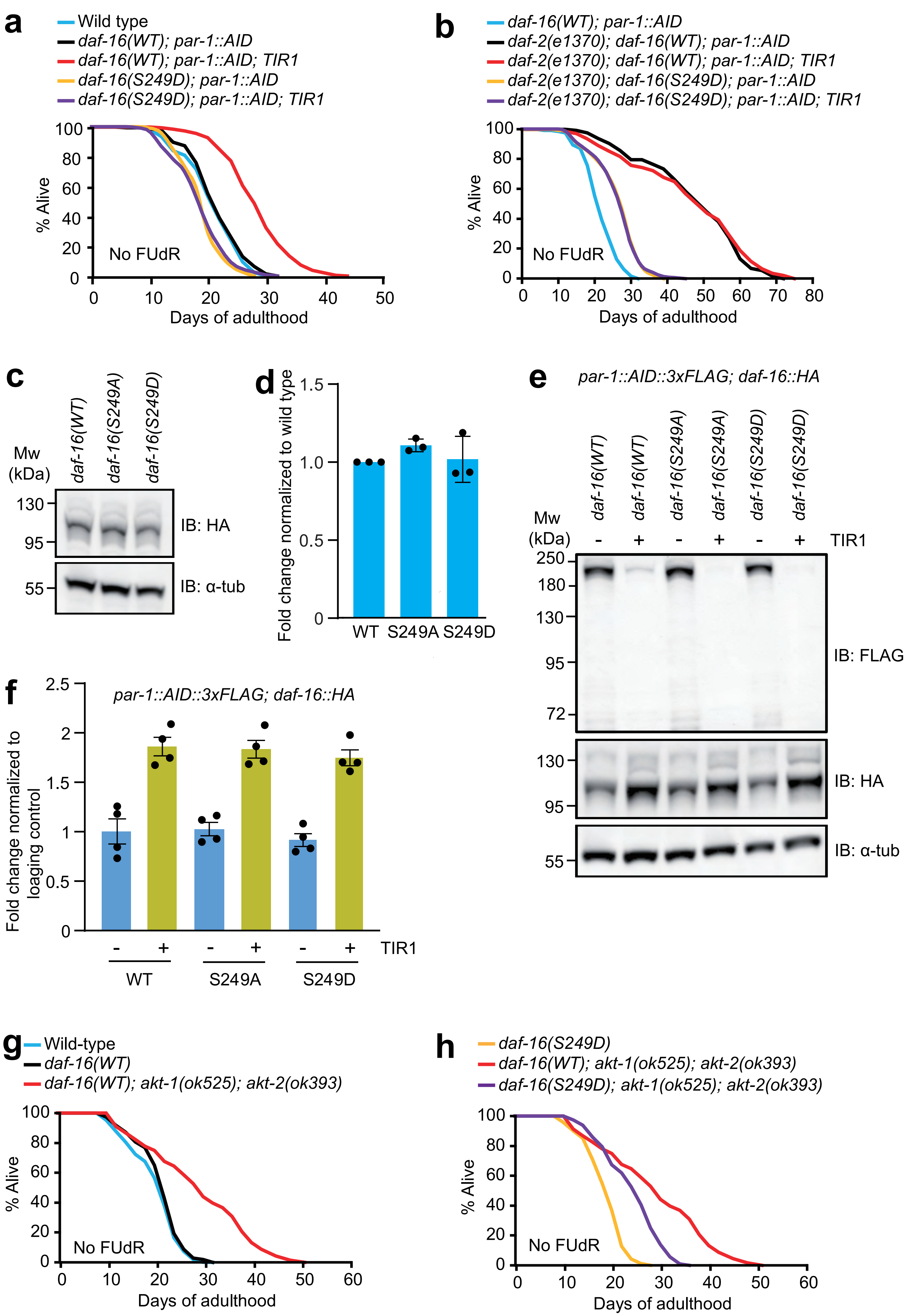

Supplementary Fig. 5. PAR-1 participates in IIS and regulates lifespan via DAF-16

a, Lifespan assays without FUdR supplementation of animals expressing *daf-16(WT)::mNG* or *daf-16(S249D)::mNG* after AID-mediated depletion of PAR-1 from young adulthood. PAR-1 knockdown increases the lifespan of animals expressing *daf-16(WT)::mNG* but not *daf-16(S249D)::mNG*.

b, Lifespan assays without FUdR supplementation of *daf-2(e1370)* mutants expressing gene-edited *daf-16(WT)::mNG* or *daf-16(S249D)::mNG* after AID-mediated depletion of PAR-1 from young adulthood. PAR-1 knockdown does not alter the lifespan of *daf-16(WT)::mNG* or *daf-16(S249D)::mNG* in a *daf-2(e1370)* background.

c, Western blot probing how the S249A and S249D substitutions alter DAF-16::mNG::HA levels. α-tubulin provides a loading control.

d, Quantification of DAF-16::HA band intensities (see panel **c**) normalized to the α-tubulin loading control. The S249A mutation marginally increased the level of DAF-16::mNG::HA compared to the wild-type control, whereas the S249D mutation had no effect. Animals were grown on normal NGM plates and harvested for Western blot analysis at L4/young adult stage (n=3 independent biological repeats).

e, Western blot probing how PAR-1 knockdown alters DAF-16(WT), DAF-16(S249A) or DAF-16(S249D) levels. PAR-1 knockdown doubles the levels of all three versions of DAF-16. α-tubulin provides a loading control.

f, Quantification of DAF-16-HA band intensities (see panel **e**) normalized to the α-tubulin loading control. These data suggest PAR-1 knockdown increases the level of

DAF-16 independently of S249 phosphorylation state. Animals were grown on NGM plates until the early L3 stage and then transferred to auxin-containing NGM plates overnight prior to harvesting for Western Blot analysis (n=4 independent biological repeats).

g, Lifespan assays without FUdR supplementation of wild-type (N2) and gene-edited animals expressing *daf-16(WT)::mNG* in a wild-type or *akt-1(ok525); akt-2(ok393)* double mutant background.

h, Lifespan assays without FUdR supplementation of gene-edited animals expressing *daf-16(WT)::mNG* or *daf-16(S249D)::mNG* in an *akt-1(ok525); akt-2(ok393)* double mutant background. Note that data for *daf-16(WT); akt-1(ok525); akt-2(ok393)* are the same as in **g**.

See Supplementary Table 6 for values and statistical analyses for lifespan assays.

Source data are provided as a Source Data file.

Supplementary Fig. 6

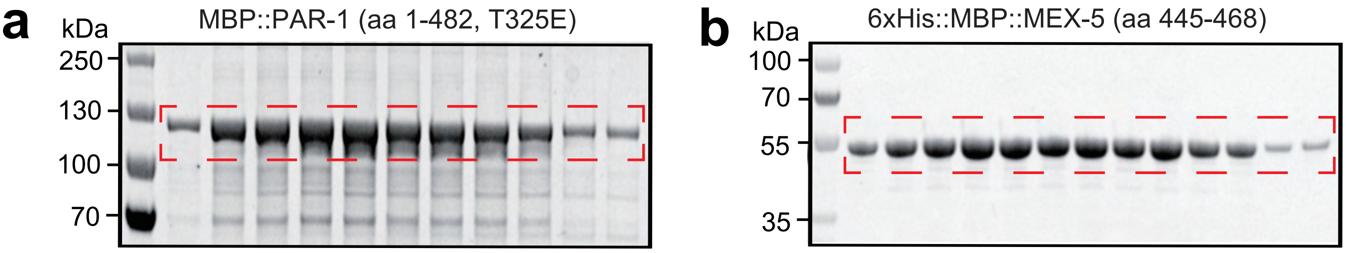

Supplementary Fig. 6. Affinity-purified recombinant PAR-1 and MEX-5

a, b, Coommassie-stained gels showing recombinant *C. elegans* PAR-1(aa 1-482, T325E, isoform a) (**a**), and MEX-5 (aa 445-468) (**b**). Proteins were expressed in *E. coli* and purified by affinity purification chromatography followed by size exclusion chromatography. Purified proteins are highlighted with dashed boxes.

Source data are provided as a Source Data file.

Supplementary Table 1, related to Fig. 2. Lifespan data analysis of *zfh-2::mSc::AID* transgenic animals on auxin-supplemented plates in wild type, *daf-16(mgDf50)* or *daf-2(e1370)* backgrounds.

Strain/treatment	Mean lifespan ±s.e.m. (days)	75th percentile	Number of animals that died/total	*p* value vs. control	Figure in text
Wild-type (N2)	22.4 ± 0.4	24	115/160		Fig. 2i
zfh-2::mSc::AID	22.5 ± 0.5	26	119/160	0.2764^{N2}	Fig. 2i
zfh-2::mSc::AID; eft-3p::TIR1::BFP	21.4 ± 0.3	24	114/160	$<0.01^{z2}$	Fig. 2i
daf-16(mgDf50); zfh-2::mSc::AID	15.0 ± 0.3	18	111/160	$<0.0001^{z2}$	Fig. 2i
daf-16(mgDf50); zfh-2::mSc::AID; eft-3p::TIR1::BFP	14.7 ± 0.3	16	90/160	$<0.0001^{z2}$ (0.4476^{z2d16})	Fig. 2i
daf-2(e1370); zfh-2::mSc::AID	50.3 ± 1.0	59	151/160	$<0.0001^{z2}$	Fig. 2h
daf-2(e1370); zfh-2::mSc::AID; eft-3p::TIR1::BFP	30.3 ± 0.7	38	119/160	$<0.0001^{z2}$ ($<0.0001^{z2d2}$)	Fig. 2h
daf-16(mgDf50); daf-2(e1370); zfh-2::mSc::AID	15.8 ± 0.4	18	91/160	$<0.0001^{z2d2}$	Fig. 2h

daf-16(mgDf50); daf-2(e1370); zfh-2::mSc::AID; eft-3p::TIR1::BFP	16.1 ± 0.3	18	107/160	$<0.0001^{z2}$ ($<0.05^{z2d2d16}$)	Fig. 2h
Wild-type (N2)	24.4 ± 0.4	28	123/160		
zfh-2::mSc::AID	25.8 ± 0.5	30	135/160	$<0.001^{N2}$	
zfh-2::mSc::AID; eft-3p::TIR1::BFP	24.0 ± 0.4	28	102/160	0.0001^{z2}	
daf-16(mgDf50); zfh-2::mSc::AID	15.9 ± 0.2	18	126/160	$<0.0001^{z2}$	
daf-16(mgDf50); zfh-2::mSc::AID; eft-3p::TIR1::BFP	16.3 ± 0.3	18	99/160	$<0.0001^{z2}$ (0.3022^{z2d16})	
daf-2(e1370); zfh-2::mSc::AID	50.0 ± 1.1	59	139/160	$<0.0001^{z2}$	
daf-2(e1370); zfh-2::mSc::AID; eft-3p::TIR1::BFP	31.2 ± 1.0	38	95/160	$<0.0001^{z2}$ ($<0.0001^{z2d2}$)	
daf-16(mgDf50); daf-2(e1370); zfh-2::mSc::AID	16.19 ± 0.3	18	119/160	$<0.0001^{z2d2}$	
daf-16(mgDf50); daf-2(e1370); zfh-2::mSc::AID; eft-3p::TIR1::BFP	17.1 ± 0.4	18	105/160	$<0.0001^{z2}$ ($0.7216^{z2d2d16}$)	

All lifespan experiments were conducted on auxin-containing plates starting from L4/YA stage.

[N2]: *p* value against N2 wild type

[z2]: *p* value against *zfh-2::mSc::AID*

[z2d16]: *p* value against *daf-16(mgDf50); zfh-2::mSc::AID*

[z2d2]: *p* value against *daf-2(e1370); zfh-2::mSc::AID*

[z2d2d16]: *p* value against *daf-16(mgDf50); daf-2(e1370); zfh-2::mSc::AID*

Supplementary Table 2, related to Fig. 3. Lifespan data analysis of *par-1::mSc::AID* transgenic animals on auxin plates in wild type, *daf-16(mgDf50)* or *daf-2(e1370)* backgrounds.

Strain/treatment	Mean lifespan ±s.e.m. (days)	75th percentile	Number of animals that died/total	*p* value vs. control	Figure in text
N2	22.8 ± 0.4	26	128/160		
par-1::mSc::AID	23.5 ± 0.5	26	127/160	0.105 [N2]	
par-1::mSc::AID; eft-3p::TIR1::BFP	27.5 ± 0.5	30	129/160	<0.0001 [p1]	

daf-16(mgDf50); par-1::mSc::AID	15.4 ± 0.2	18	120/160	<0.0001 [p1]	
daf-16(mgDf50); par-1::mSc::AID; eft-3p::TIR1::BFP	16.4 ± 0.2	18	147/160	<0.0001 [p1] (<0.01 [d16p1])	
N2	22.0 ± 0.5	24	87/120		Fig. 3j
par-1::mSc::AID	22.5 ± 0.4	26	135/160	0.3569 [N2]	Fig. 3j,k
par-1::mSc::AID; eft-3p::TIR1::BFP	29.9 ± 0.6	34	131/160	<0.0001 [p1]	Fig. 3j,k
daf-16(mgDf50); par-1::mSc::AID	16.5 ± 0.3	18	120/160	<0.0001 [p1]	Fig. 3j
daf-16(mgDf50); par-1::mSc::AID; eft-3p::TIR1::BFP	16.3 ± 0.3	18	148/160	<0.0001 [p1] (0.7792 [d16p1])	Fig. 3j
daf-2(e1370); par-1::mSc::AID	45.6 ± 0.9	53	153/160	<0.0001 [p1]	Fig. 3k
daf-2(e1370); par-1::mSc::AID; eft-3p::TIR1::BFP	45.9 ± 1.1	56	128/160	<0.0001 [p1] (0.4709 [d2p1])	Fig. 3k

All lifespan experiments for *par-1::mSc::AID* transgenic animals were conducted on auxin-containing plates starting from L4/YA stage.

[N2]: *p* value against N2 wild type

[p1]: *p* value against *par-1::mSc::AID*

[d16p1]: *p* value against *daf-16(mgDf50); par-1::mSc::AID*

[d2p1]: *p* value against *daf-2(e1370); par-1::mSc::AID*

Supplementary Table 3, related to Fig. 3. Lifespan data analysis of N2 or *daf-16(mgDf50)* worms treated with DMSO or 20 μM of compound 39621.

**Strain/treatment**	**Mean lifespan ±s.e.m. (days)**	**75th percentile**	**Number of animals that died/total**	***p* value vs. control**	**Figure in text**
N2 DMSO	20.2 ± 0.6	22	67/160		Fig. 3I
daf-16(mgDf50) DMSO	16.6 ± 0.3	18	96/160	<0.0001[N2DMSO]	Fig. 3I
N2 39621	24.6 ± 0.6	28	95/160	<0.0001[N2DMSO]	Fig. 3I
daf-16(mgDf50) 39621	16.7 ± 0.3	20	113/160	0.8642[d16DMSO]	Fig. 3I
N2 DMSO [#]	20.3 ± 0.5	24	109/160		
daf-16(mgDf50) DMSO [#]	16.0 ± 0.3	18	118/160	<0.0001[N2DMSO]	
N2 39621 [#]	23.8 ± 0.6	28	128/160	<0.0001[N2DMSO]	

daf-16(mgDf50) 39621 #	16.8 ± 0.3	18	146/160	<0.05 d16DMSO	
N2 DMSO #	23.6 ± 0.5	26	89/160		
daf-16(mgDf50) DMSO #	15.8 ± 0.3	18	111/160	<0.0001 N2DMSO	
N2 39621 #	25.9 ± 0.5	28	124/160	0.001 N2DMSO	
daf-16(mgDf50) 39621 #	16.2 ± 0.3	20	149/160	0.3891 d16DMSO	

All lifespan experiments were conducted on normal NGM plates.

N2DMSO: *p* value against N2 wild type treated with DMSO

d16DMSO: *p* value against *daf-16(mgDf50)* mutants treated with DMSO

#: lifespan without FUdR-treated conditions

Supplementary Table 4, related to Fig. 4 and Supplementary Fig. 4. Lifespan data analysis of *daf-16(WT)::mNG::HA*, *daf-16(S249A)::mNG::HA* and *daf-16(S249D)::mNG::HA* transgenic worms in wild-type or *daf-2(e1370)* mutant background.

Strain/treatment	Mean lifespan ±s.e.m. (days)	75th percentile	Number of animals	*p* value vs. control	Figure in text

			that died/total		
N2	21.5 ± 0.5	24	93/120		Fig. 4g
daf-16(WT)::mNG::HA	22.0 ± 0.4	26	130/160	0.227 ^{N2}	Fig. 4g,h
daf-16(S249A)::mNG::HA	23.9 ± 0.4	28	139/160	<0.01 ^{WT}	Fig. 4g
daf-16(S249D)::mNG::HA	19.8 ± 0.3	22	118/160	<0.0001 ^{WT}	Fig. 4g
daf-2(e1370); daf-16(WT)::mNG::HA	48.1 ± 1.2	59	144/160	<0.0001 ^{WT}	Fig. 4h
daf-2(e1370); daf-16(S249A)::mNG::HA	47.7 ± 1.2	59	144/160	<0.0001 ^{WT} (0.4044 ^{d2WT})	Fig. 4h
daf-2(e1370); daf-16(S249D)::mNG::HA	28.3 ± 0.9	35	97/120	<0.0001 ^{WT} (<0.0001 ^{d2WT})	Fig. 4h
N2 $^{\#}$	20.4 ± 0.3	24	122/160		Supplementary Fig. 4c
daf-16(WT)::mNG::HA $^{\#}$	20.4 ± 0.3	22	127/160	0.8664 ^{N2}	Supplementary Fig. 4c,d

daf-16(S249A)::mNG::HA #	22.7 ± 0.4	26	115/160	<0.0001 WT	Supplementary Fig. 4c
daf-16(S249D)::mNG::HA #	18.6 ± 0.3	20	114/160	<0.0001 WT	Supplementary Fig. 4c
daf-2(e1370); daf-16(WT)::mNG::HA #	49.1 ± 1.3	60	118/160	<0.0001 WT	Supplementary Fig. 4d
daf-2(e1370); daf-16(S249A)::mNG::HA #	51.7 ± 1.3	60	108/160	<0.0001 WT (0.1642 d2WT)	Supplementary Fig. 4d
daf-2(e1370); daf-16(S249D)::mNG::HA #	26.7 ± 0.7	33	120/160	<0.0001 WT (<0.0001 d2WT)	Supplementary Fig. 4d

All lifespan experiments were conducted on normal NGM plates (without auxin).

N2: *p* value against N2 wild type

WT: *p* value against *daf-16(WT)::mNG::HA*

d2WT: *p* value against *daf-2(e1370); daf-16(WT)::mNG::HA*

#: lifespan without FUdR-treated conditions

Supplementary Table 5, related to Fig. 5 and Supplementary Fig. 5. Lifespan data analysis of *par-1::mSc::AID* transgenic animals in the presence or absence of *TIR1* on auxin plates in wild-type or *daf-2(e1370)* backgrounds and expressing WT or S249D form of DAF-16.

Strain/treatment	Mean lifespan ±s.e.m. (days)	75th percentile	Number of animals that died/total	*p* value vs. control	Figure in text
N2	24.2 ± 0.4	28	126/160		Fig. 5a
daf-16(WT)::mNG::HA; par-1::mSc::AID	23.8 ± 0.3	26	118/160	0.1453 ^{N2}	Fig. 5a,b
daf-16(WT)::mNG::HA; par-1::mSc::AID; eft-3p::TIR1::BFP	28.9 ± 0.5	32	119/160	<0.01 ^{WTp1}	Fig. 5a
daf-16(S249D)::mNG::HA; par-1::mSc::AID	19.9 ± 0.4	22	99/160	<0.0001 ^{WTp1}	Fig. 5a
daf-16(S249D)::mNG::H	20.0 ± 0.3	22	138/160	<0.0001 ^{WTp1}	Fig. 5a

A; par-1::mSc::AID; eft-3p::TIR1::BFP				(0.9017^{S249Dp1})	
daf-2(e1370); daf-16(WT)::mNG::HA; par-1::mSc::AID	48.6 ± 1.0	59	154/160	<0.0001 ^{WTp1}	Fig. 5b
daf-2(e1370); daf-16(WT)::mNG::HA; par-1::mSc::AID; eft-3p::TIR1::BFP	48.6 ± 1.1	59	132/160	<0.0001^{WT} (0.8828 ^{d2WTp1})	Fig. 5b
daf-2(e1370); daf-16(S249D)::mNG::HA; par-1::mSc::AID	24.3 ± 0.5	29	88/120	<0.0001 ^{d2WTp1}	Fig. 5b
daf-2(S249D); daf-16(WT)::mNG::HA; par-1::mSc::AID; eft-3p::TIR1::BFP	25.8 ± 0.4	29	113/120	<0.0001 ^{d2WTp1} (<0.05^{d2S249Dp1})	Fig. 5b
N2 #	21.1 ± 0.4	24	160/200		Supplementary Fig. 5a
daf-16(WT)::mNG::HA; par-1::mSc::AID #	21.8 ± 0.4	26	123/160	0.2377 ^{N2}	Supplementary Fig. 5a,b
daf-16(WT)::mNG::HA;	28.8 ± 0.4	32	178/200	<0.0001 ^{WTp1}	Supplementary Fig. 5a

par-1::mSc::AID; eft-3p::TIR1::BFP #					
daf-16(S249D)::mNG::HA; par-1::mSc::AID #	19.3 ± 0.3	22	120/160	<0.0001 *WTp1*	Supplementary Fig. 5a
daf-16(S249D)::mNG::HA; par-1::mSc::AID; eft-3p::TIR1::BFP #	19.1 ± 0.4	22	153/160	<0.0001 *WTp1* (0.6954*S249Dp1*)	Supplementary Fig. 5a
daf-2(e1370); daf-16(WT)::mNG::HA; par-1::mSc::AID #	47.8 ± 1.2	60	128/200	<0.0001 *WTp1*	Supplementary Fig. 5b
daf-2(e1370); daf-16(WT)::mNG::HA; par-1::mSc::AID; eft-3p::TIR1::BFP #	47.0 ± 1.4	60	137/200	<0.0001*WT* (0.5515 *d2WTp1*)	Supplementary Fig. 5b
daf-2(e1370); daf-16(S249D)::mNG::HA; par-1::mSc::AID #	27.2 ± 0.5	33	140/200	<0.0001 *d2WTp1*	Supplementary Fig. 5b
daf-2(e1370); daf-16(S249D)::mNG::HA; par-1::mSc::AID; eft-3p::TIR1::BFP #	27.3 ± 0.5	30	145/160	<0.0001 *d2WTp1* (0.9425 *d2S249Dp1*)	Supplementary Fig. 5b

All lifespan experiments were conducted on auxin-containing plates starting from L4/YA stage.

^{N2}: p value against N2 wild type

^{WTp1}: p value against *daf-16(WT)::mNG::HA; par-1::mSc::AID*

^{S249Dp1}: p value against *daf-16(S249D)::mNG::HA; par-1::mSc::AID*

^{d2WTp1}: p value against *daf-2(e1370); daf-16(WT)::mNG::HA; par-1::mSc::AID*

^{d2S249Dp1}: p value against *daf-2(e1370); daf-16(S249D)::mNG::HA; par-1::mSc::AID*

$^{\#}$: lifespan without FUdR-treated conditions

Supplementary Table 6, related to Fig. 5 and Supplementary Fig. 5. Lifespan data analysis of *daf-16(WT)::mNG::HA* or *daf-16(S249D)::mNG::HA* transgenic worms in *akt-1(ok525); akt-2(ok393)* double mutant background.

**Strain/treatment**	**Mean lifespan ±s.e.m. (days)**	**75th percentile**	**Number of animals that died/total**	***p* value vs. control**	**Figure in text**
N2	22.8 ± 0.5	26	99/160		Fig. 5c
daf-16(WT)::mNG::HA	23.9 ± 0.4	26	125/160	0.0966 ^{N2}	Fig. 5c

daf-16(S249D)::mNG::HA	19.4 ± 0.4	22	82/160	<0.0001 *WT*	Fig. 5d
daf-16(WT)::mNG::HA; akt-1(ok525); akt-2(ok393)	35.8 ± 1.3	51	147/160	<0.0001 *WT*	Fig. 5c,d
daf-16(S249D)::mNG::HA; akt-1(ok525); akt-2(ok393)	23.8 ± 0.6	28	104/160	<0.0001 *S249D* (<0.0001 *WTa1a2*)	Fig. 5d
N2 #	20.5 ± 0.5	24	110/160		Supplementary Fig. 5g
daf-16(WT)::mNG::HA #	21.5 ± 0.5	24	104/160	0.2149 *N2*	Supplementary Fig. 5g
daf-16(S249D)::mNG::HA #	19.0 ± 0.4	22	97/160	<0.0001 *WT*	Supplementary Fig. 5h
daf-16(WT)::mNG::HA; akt-1(ok525); akt-2(ok393) #	29.3 ± 1.3	38	68/160	<0.0001 *WT*	Supplementary Fig. 5g,h
daf-16(S249D)::mNG::HA;	24.5 ± 0.6	28	109/160	<0.0001 *S249D*	Supplementary Fig. 5h

akt-1(ok525); akt-2(ok393) #				(<0.0001 ^{WTa1a2})	

All lifespan experiments were conducted on normal NGM plates (without auxin).

^{N2}: *p* value against N2 wild-type

^{WT}: *p* value against *daf-16(WT)::mNG::HA*

^{S249D}: *p* value against *daf-16(S249D)::mNG::HA*

^{WTa1a2}: *p* value against *daf-16(WT)::mNG::HA; akt-1(ok525); akt-2(ok393)*

#: lifespan without FUdR-treated conditions

Supplementary Table 6, related to Fig. 5. Lifespan data analysis of *par-1(WT)::mEGFP*, *par-1(KRSS)::mEGFP*, *par-1(ΔKA1)::mEGFP* and *par-1(T983A)::mEGFP* transgenic worms.

Strain/treatment	Mean lifespan ±s.e.m. (days)	75th percentile	Number of animals that died/total	*p* value vs. control	Figure in text
N2 #	22.5 ± 0.5	26	94/160		
par-1(WT)::mEGFP #	20.9 ± 0.5	24	118/160	<0.05^{N2}	Fig. 5e,f

par-1(KRSS)::mEGFP #	17.8 ± 0.5	22	106/160	0.0001^{WT}	Fig. 5f
par-1(ΔKA1)::mEGFP #	16.0 ± 0.4	20	100/160	<0.0001^{WT}	Fig. 5f
par-1(T983A)::mEGFP #	14.4 ± 0.4	18	98/160	<0.0001^{WT}	Fig. 5e
N2 #	20.1 ± 0.5	24	136/160		
par-1(WT)::mEGFP #	20.1 ± 0.5	24	130/160	0.8673^{N2}	
par-1(KRSS)::mEGFP #	18.8 ± 0.4	22	129/160	<0.05^{WT}	
par-1(ΔKA1)::mEGFP #	17.0 ± 0.4	20	121/160	<0.0001^{WT}	
par-1(T983A)::mEGFP #	16.0 ± 0.4	20	99/160	<0.0001^{WT}	

All lifespan experiments were conducted on normal NGM plates (without auxin).

^{N2}: *p* value against N2 wild type

^{WT}: *p* value against *par-1(WT)::mEGFP*

$^{\#}$: lifespan without FUdR-treated conditions
