# Supplementary material for: Proximity labeling of DAF-16 FOXO highlights aging regulatory proteins
Source: Nat Commun. 2025 Dec 11;16:11355. doi: 10.1038/s41467-025-66409-0 (PMC12727705; doi:10.1038/s41467-025-66409-0)
Supplement: Supplementary file 2 — Description of Additional Supplementary Files [file 41467_2025_66409_MOESM2_ESM.pdf]

**Title:** Supplementary Data 1

**Description:** Mass spectrometry data for proximity labeling of DAF-16 in wild type and daf-2 mutant backgrounds

**Title:** Supplementary Data 2

**Description:** Comparison of proximity labeling data for DAF-16 with interactors identified in earlier studies

**Title:** Supplementary Data 3

**Description:** Mass spectrometry data assessing DAF-16 phosphorylation in different genetic backgrounds

**Title:** Supplementary Data 4

**Description:** List of C. elegans strains used in this study

**Title:** Supplementary Data 5

**Description:** List of crRNAs and primers used in this study for CRISPR editing and qPCR
